# What are the predictors and costs of nurse absenteeism at select multicenter government hospitals? A cross-sectional study

**DOI:** 10.3389/fpubh.2023.1073832

**Published:** 2023-02-27

**Authors:** Hashem Al Ismail, Nawal H. Herzallah, Sultan T. Al-Otaibi

**Affiliations:** ^1^Qatif Central Hospital, Ministry of Health, Dammam, Saudi Arabia; ^2^Imam Abdulrahman Bin Faisal University, Department of Public Health, College of Public Health, Dammam, Saudi Arabia

**Keywords:** predictors, cost, nurse, absenteeism, occupational

## Abstract

**Objectives:**

The purposes of this study were to determine the prevalence and cost of absenteeism in nurses as well as the factors that affect absenteeism.

**Methods:**

This is a cross sectional study where a self-administered questionnaire response were obtained from 442 nurses for the previous working in 4 hospitals and 3 primary health care centers in Saudi Arabia. Analyses compared those with zero absences with those with one or more absences per month. Attributable risk was calculated as the difference in the absence percentages among nurses with high-risk exposure and low risk exposure.

**Results:**

The average absence of nurses is 0.62 days per month. This results in an annual loss of around $4 million. The greatest absence frequency was significantly associated with work psychosocial factors. The modifiable factors included the clarity of work responsibilities, rating of managers, work facilities, work environment, transportation difficulties, and work satisfaction. Cost-effectiveness modules for absence intervention programs were built for these factors.

**Conclusions:**

This study demonstrated that nurse absenteeism is a costly issue related to work and psychosocial factors. Preventive programs to improve the quality of work life are likely to be cost effective.

## Introduction

Nurses play a central role in frontline patient care. They are the largest group of health care professionals in Saudi Arabia (1). Nurse absenteeism is frequent, costly, and affects work productivity (2–4). It has significant impacts on the quality of patient care and staffing instability (5–8).

Canadian studies showed that the “adequacy of nursing staffing and proportion of registered nurses are inversely related to the death rate of acute medical patients within 30 days of hospital admission” (9, 10). Another study reported that a 10% increase in the number of patients assigned to a nurse leads to a 28% increase in adverse health events such as infections, medication errors, and other injuries (11).

Absenteeism can be defined as not coming to work when scheduled. Numerically, absenteeism equals the “sum of the periods when the employees of a given organization are absent from work, as opposed to absence motivated by unemployment, prolonged disease or a legal leave from work” (12).

Total days away from work may be due to personal illness, or scheduled vacations. However, absences may be caused by unexpected and otherwise unexplained reasons. The category of unanticipated volitional absence is likely to include potentially preventable absences (13–15). A systematic review showed that the following factors reduce absenteeism: job satisfaction, organizational commitment, attendance records, involvement, and retention factors. Burnout and job stress were shown to increase absenteeism (8).

While many studies focus on health care absenteeism worldwide, few focus on nursing staffs and predictors of absenteeism in Saudi Arabia. A sample of 405 nurses working at Medina in Saudi Arabia indicated that the most common predictive factors associated with absenteeism were a lack of overtime payment (75.6%) and social reasons among 77.8% of nurses (16). Another study was conducted among 110 nurses working in Hail, Saudi Arabia with a majority of them male, married, and aged 35–39 years. The factors influencing absenteeism included health problems (40%), the working environment (24.5%), and personal and family problems (24.5%) (17). Absenteeism showed no relationship with the work environment among Swiss nursing homes (18).

This study focuses on absenteeism of nursing staff working in the eastern province of Saudi Arabia, and identifies factors associated with absenteeism. Our objectives were to:

- Estimate the prevalence of absenteeism among nurses,
- Analyze the direct cost of absenteeism among nurses, and
- Analyze the factors that affect absenteeism among nurses.

## Methods

### Study setting and design

A cross-sectional study was designed to measure the factors associated with absenteeism among nursing staffs in the Eastern Province of Saudi Arabia. The data were collected using a self-administered questionnaire (paper based) designed by the authors. Three research supervisors at the Imam Abdulrahman Bin Faisal University of Saudi Arabia validated the contents of the questionnaire, and its concurrent validity.

A summary rating scale of psychosocial predictors with reported absence (work environment, clarity of responsibilities, work satisfaction, rating of managers and relation type with co-workers) was used, with four choices per item as follows: (1) *excellent*, (2) *good*, (3) *fair*, (4) and *don't know*. The internal consistency of the overall scale of the predictors was assessed using Cronbach's alpha with a 95% confidence interval (CI), and it was determined to be good (Cronbach's alpha = 0.83; 95% CI: 0.80–0.86). This indicates that the instrument was reliable in measuring what it should.

The items in the questionnaire included (a) personal factors (e.g., age, gender, nationality, marital status, number of children, and availability of transportation to work); (b) work factors [e.g., job title, years of experience, work area (department), work facilities (work environment), rating, and shift work]; (c) work psychosocial factors (e.g., work satisfaction, relation to coworkers, and superior rating); (d) absences (number in the past 30 days, total days, sickness, and other causes); and (e) absence causes, consequences, and work pressure factors (according to the nurses).

Further, documents of absenteeism were reviewed from three main hospitals (Dammam, Qatif, and Dhahran).

The sample size was estimated using EpiInfo ver7. A minimum sample size of 420 participants was required for a 95% confidence interval with a power of the study of 80%.

#### Inclusion criteria

Any nurse in direct contact with patients and having signed a job contract with the Ministry of Health was eligible to participate in the study.

#### Exclusion criteria

Nurse interns, students, trainees, nurses working in administrative jobs, and nurses with < 1 year of experience were excluded from the study.

### Data collection

A cluster sample (convenience sample) of 476 nurses was recruited from four different hospitals and three primary health care centers (PHCs). Data were collected from April to June 2017. Thirty-four nurses were excluded since they had administrative jobs, or interns, or students, or trainees or <1 year of work experience.

The questionnaire asked each nurse for an estimate of the average number of absences per month; these absences were described as “reported absences” which was used as an outcome variable of absenteeism. More detailed information is available for three participating hospitals (Dammam, Qatif, and Dhahran) in which personnel records are available for review.

Preventive programs for selected modifiable risk factors were recommended (e.g., public transportation, pre-employment work description, a manager training program, work environment improvement) in which a hypothetical predicted cost of each preventive program was estimated. Then, the net benefit value was calculated by subtracting the attributable cost of each absence factor from the predicted cost of each preventive program with multiple predicted effectiveness. Cost-effectiveness modules were built based on the results to estimate the benefit of each program.

### Data analysis

The data were entered and analyzed using IBM SPSS Statistics version 22.0 (SPSS Inc., Chicago, Ill., USA). The analysis compared those with zero absences with those with one or more absences per month. Reported absences was used as an outcome variable of absenteeism. Frequencies and percentages were calculated for the categorical variables, and the associations were assessed using Chi squares. Multivariate model that compares the factors that may predict absenteeism was done. We investigated the results of the logistic regression analysis to evaluate the relationships among multiple factors, assuming linearity and that the observations were independent of one another. A *p*-value of < 0.05 was considered as statistically significant.

The direct annual absence cost in US dollars was calculated by multiplying the total number of nurses in the Eastern Province by the mean absence per nurse per month for twelve months by the mean salary divided by working day of the nurses. We also calculated the attributable risk for each factor as the difference between the absence percentages of nurses with high-risk predictor factors and those with low risk predictor factors. The attributable risk of each absence factor was multiplied by the reported absence cost to calculate the attributable cost.

### Ethical considerations

The study protocol was approved by the Ethical Committee of the General Directorate of Health Affairs in the Eastern Province and also by Imam Abdulrahman Bin Faisal University Ethical Committee, written consent was obtained from each participant. All the procedures involving human participants were conducted in accordance with the ethical standards of the institutional and/or national research committee and with the 1964 Helsinki Declaration and its later amendments or comparable ethical standards.

## Results

442 nurses (92% females and 8% males) were included in the analysis of this study. The majority (76%) were Saudi, while the remaining (24%) were non-Saudi. Most of the nurses (53%) were 26–30 years old and married (78%). Sixty-seven percent of nurses were assistants or technicians, and the majority (53%) worked either in wards or in emergency rooms.

Table 1 summarizes the personal factors associated with reported absences. Younger nurses (aged 20–25 years) were frequently absent. Among those aged at least 30 years, only 22% were absent, whereas 59% of those under the age of 25 years were more frequently absent within the past month (*P* < 0.001). Nurses of Saudi nationality were considerably more likely to be absent than non-Saudi nurses (40 vs. 9%, *P* < 0.001).

**Table 1:** Personal factors associated with absence.

**Variable**		***N***	**Absence (days/month)**				**χ^2^ *p*-value**	**Attributable risk**
		**0**		≥**1**				
**Age**	20–25	59	24	41%	35	59%	<0.001	37%
	26–30	234	152	65%	82	35%		
	>30	149	117	78%	32	22%		
**Nationality**	Saudi	335	201	60%	134	40%	<0.001	31%
	Non-Saudi	107	97	91%	10	9%		
**Transportation difficulties**	Yes	212	122	58%	90	42%	<0.001	17%
	No	230	172	75%	58	25%
**Gender**	Male	36	22	61%	14	39%	0.26	7%
	Female	406	272	67%	134	33%		
**Marital Status**	Single	107	76	71%	31	29%	0.26	6%
	Married	335	217	65%	118	35%		
**Children**	0	100	66	66%	34	34%	0.47	0%
	≥1	335	221	66%	114	34%		

The other personal factors included in Table 1, namely gender, marital status, and number of children, were not associated with the absences (the *P*-values were statistically insignificant).

The work factors associated with reported absences are illustrated in Table 2. Less educated (with a diploma in nursing) and less experienced nurses were considerably more likely to be absent from work. For example, 40% of nurse technicians (diploma in nursing) were absent compared to 26% of nurse specialists (with a bachelor's degree in nursing; *P* < 0.001). Moreover, nurses with at least 10 years of experience were absent only 19% of the time, whereas those with <5 years of experience were absent 45% of the time (*P* < 0.001). The emergency departments showed the highest rate of absenteeism (55%; *P* = 0.02). Quality of work facilities contributed significantly to absenteeism (*P* < 0.001). Nurses with excellent facilities were absent 25% of the time, while those with fair facilities were absent 29% of the time (*P* < 0.001). Shift work and absence policy were not statistically significantly associated with absenteeism.

**Table 2:** Work factors associated with absence.

**Variable**		***N***	**Absence (days/month)**				**χ^2^ *p*-value**	**Attributable risk**
		**0**		≥**1**				
**Job title**	Nurse assistant	44	27	62%	17	38%	<0.001	25%
	Nurse technician	245	147	60%	98	40%		
	Nurse specialist	146	108	74%	38	26%		
	Nurse senior specialist	7	7	100%	0	0%		
**Experience (by year)**	1–5	196	108	55%	88	45%	<0.001	26%
	6–10	129	90	70%	39	30%		
	>10	117	95	81%	22	19%		
**Work area**	Ward	194	122	63%	72	37%	<0.001	23%
	Clinic	89	61	68%	28	32%		
	Emergency Room	48	22	45%	26	55%		
	Other	111	90	81%	21	19%		
**Work facilities rating**	Excellent	43	32	75%	11	25%	<0.001	4%
	Good	175	121	69%	54	31%		
	Fair	161	114	71%	47	29%		
	Do not know	63	27	43%	36	57%		
**Working time**	Shift	282	181	64%	101	36%	0.27	7%
	Fixed time	160	114	71%	46	29%		
**Type of absence policy**	Excellent	29	19	67%	10	33%	0.31	3%
	Good	101	63	62%	38	38%		
	Fair	151	97	64%	54	36%		
	Do not know	161	113	70%	48	30%		

Work environment, clarity of responsibility, work satisfaction, and facility rating were significantly associated with nurse absenteeism (Table 3). Nurses with an excellent work environment tended to have a lower rate of absenteeism (23%) than those with a fair environment (35%; *P* < 0.001). Degree of clarity with regard to work responsibilities also had a large impact; only 18% of nurses with well-defined responsibilities were absent from work, whereas this rate was as high as 52% for those with fairly defined responsibilities (*P* < 0.001). Nurses who were very satisfied with work were absent less frequently (16%) than those who were fairly satisfied with work (45%; *P* < 0.001). Organizational factors contributed significantly to absenteeism. Nurses who liked their managers showed lower rates of absenteeism (24%) than those who disliked their managers (42%; *P* < 0.001). Relations with coworkers had little impact (*P* = 0.12).

**Table 3:** Psychosocial factors associated with absence.

**Variable**		***N***	**Absence (days/month)**				**χ^2^ *p*-value**	**Attributable risk**
			**0**		≥**1**			
**Work environment**	Excellent	44	34	77%	10	23%	<0.001	12%
	Good	174	127	73%	47	27%		
	Fair	164	107	65%	57	35%		
	Do not know	60	29	48%	31	51%		
**Clarity of responsibilities**	Very well clear	148	121	82%	27	18%	<0.001	34%
	Well clear	150	101	67%	49	33%		
	Fairly clear	128	61	48%	67	52%		
	Do not know	16	11	69%	5	31%		
**Work satisfaction**	Very well satisfied	86	72	84%	14	16%	<0.001	29%
	Well satisfied	175	124	71%	51	29%		
	Fairly satisfied	132	73	55%	59	45%		
	Do not know	49	24	49%	25	51%		
**Rating of managers**	Excellent	123	93	76%	30	24%	<0.001	18%
	Good	187	125	67%	62	33%		
	Fair	108	63	58%	45	42%		
	Do not know	24	11	46%	13	54%		
**Relation type with co-workers**	Excellent	169	117	69%	52	31%	0.12	10%
	Good	219	147	67%	72	33%		
	Fair	51	30	59%	21	41%		
	Do not know	3	0	0%	3	100%		

Multiple logistic regression modules (Table 4) were implemented for the variables thought to be affected by nationality. The *p*-values for work satisfaction, rating of managers, and clarity of work responsibilities were < 0.05. This means that these variables are not confounders for absenteeism. However, the *p-*value for transport difficulties and work environment exceeded 0.05, indicating that they are cofounders in association with absenteeism.

**Table 4:** Multiple logistic modules for different factors vs. nationality.

	**Factor**	**Estimate**	**SE**	***p*-value**	**OR**	**95% CI**
Module 1	Nationality (Saudi)					
	Work Satisfaction	0.416	0.130	0.001	1.51	1.17–1.95
Module 2	Nationality (Saudi)					
	Rating of managers	0.460	0.134	0.001	1.58	1.22–2.06
Module 3	Nationality (Saudi)					
	Transport difficulty	0.881	0.478	0.065	2.41	0.94–6.15
Module 4	Nationality (Saudi)					
	Work environment	0.258	0.135	0.056	1.29	0.99–1.68
Module 5	Nationality (Saudi)					
	Clarity of work responsibilities	0.352	0.138	0.011	1.42	1.08–1.86

SE, standard error; OR, odds ratio; CI, confidence interval.

### Perception of the causes and consequences of absenteeism

Most nurses indicated that health issues and work stress (43%) were the main causes of absence, followed by social obligations, job dissatisfaction (36%), and other factors (delayed performance, overtime, turnover). Forty-two percent of Saudi nurses indicated that social obligations were a cause of absenteeism whereas 22.0% of non-Saudi nurses felt the same way. The largest group (48%) of nurses indicated that a heavy workload was the main factor contributing to work stress, followed by supervision style (44%), improper work environment (33%), and forced work (28%). Most nurses (52%) indicated that absenteeism could be reduced through recognition of work, better work conditions, and more coordination with coworkers, followed by other factors.

### Cost of absenteeism by cause

Thirty-three percent of the nurses were absent at least one day per month. Our study found a reported mean absence rate of 0.62 days per employee per month, amounting to a total of 266 days lost per month among 442 nurses. The absence documents indicated a mean of 0.2, 0.2, and 0.9 days of absence per employee per month for Dhahran, Qatif, and Dammam, respectively, yielding an average mean of approximately 0.5 days (0.43 absences per employee per month), which is close to the mean of our study (0.62 absences per employee per month).

Considering an absence mean of 0.62, an average nurse salary of $1353 per month, 20 working days per month, and the total number of nurses in the eastern province of Saudi Arabia (7991), we calculated that nurse absenteeism costs the General Directorate of Health Affairs in the Eastern Province of Saudi Arabia ~$4,021,998 per year (19, 20).

Each absence factor contributes to the cost depending on its attributable risk value. For instance, attributable risk associated with the manager rating is 18%. Thus, attributable risk from this factor contributes $723,959 (0.18 × 4,021,998) to the total cost. The cost of each absence factor depending on its attributable risk. Quality of work is the most modifiable absence factor, and it contributes substantially to the cost. Some of the other modifiable factors are work satisfaction, clarity of work responsibilities, manager rating, and work environment. Moreover, transportation is a modifiable factor with an attributable risk of 17%.

Based on the attributable cost associated with each factor, cost-effectiveness modules were generated in accordance with the hypothetical cost and effectiveness of each program recommended to reduce absenteeism. For example, if $50 is spent on each nurse for the pre-employment job description course, assuming a predicted effectiveness of 50%, a net value of $35 will be saved annually (0.5 × 171– 50). When projected to all nurses of the eastern province (7991), $279,685 will be saved annually by modifying this single factor. According to the predicted cost-effectiveness module, the optimum effectiveness of the manager training program is 30%. This will save ~$7.5 per nurse annually, amounting to a total of $59,933.

**Figure 1:**
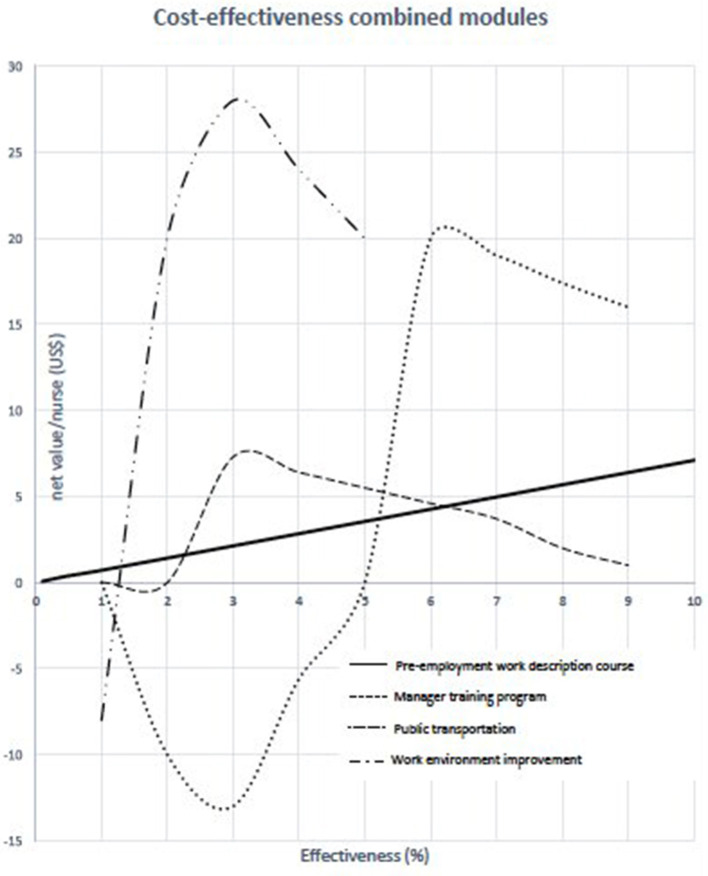
The combined cost-effectiveness modules.

Figure 1 illustrates the combined cost-effectiveness modules for all four recommended programs. The optimum effectiveness of a manager training program is 30%. This was confirmed with calculation as it would save around $7.5/nurse annually for a total of US$59.93. Also in Figure 1, the pre-employment job description course had a predicted cost-effectiveness of 50%. By contrast, public transportation and work environment improvement programs are of greater cost to achieve a benefit. Improving the work environment yields the greatest benefit but results in the lowest predicted effectiveness.

## Discussion

This study found that the average absence of nurses is 0.62 days per month. This results in an annual loss of around $4 million. The greatest absence frequency was significantly associated with work psychosocial factors while quality of work was the most modifiable factor. In addition, our study showed the importance of potentially remediable psychosocial workplace factors. This study had identified a clear relationship between nurse-supervisor rapport and the likelihood of largely volitional absences.

Our study sample was large (*n* = 442) and representative of nurses in the eastern province of Saudi Arabia. The sample represents 5.5% of the total number of nurses (7991) who work in governmental health institutes in the eastern province of Saudi Arabia. The documented rate of absence was 0.43 day per employee per month, which is very close to the mean of our study (0.62). The studied personnel records specifically identified unjustified (i.e., volitional) absences as determined by the supervisor. This distinction is not available in other published studies (21, 22).

Unlike in other studies, this study demonstrated that nurse absenteeism was not significantly associated with some personal factors, such as marital status, number of children (childcare responsibilities), and gender (being a male), that lead to higher rate of absenteeism (23–27). Our results were also consistent with those of other studies that show that absenteeism occurs more frequently among younger, less experienced, and less educated nurses (16, 17).

A very strong association was noted between absenteeism and nationality. This was probably due to the greater social obligations perceived by the Saudi nurses (42%) compared to their non-Saudi (22%) counterparts and the same finding was reported in another study (28). Another reason for absenteeism was that the hospital policy may be unfairly biased toward local nurses. This belief could be overcome by devising and implementing a strict and fair policy that is equally applied to all nurses (17, 24–26).

The quality of work life (job satisfaction, job environment, facilities, and good superiors) was significantly associated with absenteeism. This was consistent with another studies that showed “attitudes (job satisfaction, organizational commitment, and work/job involvement)” and leadership style and feeling respect from supervisors will reduced nurse absenteeism (29–32). Most nurses reported facing a heavy workload and improper work conditions, leading to greater work stress and absenteeism. Finally, most nurses noted that better work conditions and recognition of their work as well as coordination with coworkers could reduce absenteeism (30–32). All these factors signify that the quality of the work environment and demands are important predictors of absenteeism.

Our study demonstrated that the average nurse was absent 0.62 days per month, resulting in a loss of productive work of 266 days per month among the 429 nurses studied. The number of lost days per employee per year is 7.4, which is higher and costly than the international average of 7 by almost half a day (19, 20).

In this study educational programs for nurse supervisors and improvement of their communication skills had a predicted effectiveness rate is 30%. Moreover, providing pre-employment job descriptions entails minimal cost, but promises a high cost-effectiveness rate. Public transportation and work environment improvement programs may lead a higher cost with regard to achieving benefits in the first year, but their costs are likely to be discounted in subsequent years.

### Strengths of this study

- This study demonstrated that nurses' absenteeism is a costly issue related to work and psychosocial factors.
- We presented the combined cost-effectiveness modules for all recommended programs.

### Limitations of this study

- The reliability of the study may be lower than expected due to self-reported absence, however the absence documents were reviewed from three hospitals, and the mean absence was found to be close to the mean of our study.
- The use of scales in this study may be positively biased as respondents appear to have had the option to choose between *excellent, good, fair*, and *do not know*, and this may suggest that negative options may have been chosen.
- Our samples were of the cluster type and were heterogeneous but were analyzed together, which may influence the accuracy of our analysis, possibly leading to analysis bias.

## Conclusions

The study demonstrated that nurse absenteeism in the hospitals and PHCs of the studied areas was highly prevalent and costly. Nationality and quality of work factors were associated with absenteeism. Some absence predictors are modifiable and can be corrected with highly cost-effective programs such as pre-employment courses, provision of transportation, and manager training. Qualitative elements of absenteeism among nurses would have provided a lot richer data and greater depth to the study and these are proposed to consider in future research.

## Data availability statement

The raw data supporting the conclusions of this article will be made available by the authors, without undue reservation.

## Ethics statement

The studies involving human participants were reviewed and approved by the Imam Abdulrahman Bin Faisal University. The patients/participants provided their written informed consent to participate in this study.

## Author contributions

HA: collection of data, statistical analysis, and literature review. NH: literature review and manuscript editing. SA-O: study design, literature review, and writing manuscript. All authors contributed to the article and approved the submitted version.
